# Case Report: Upper gastrointestinal bleeding and multiorgan injury caused by ethylicin poisoning

**DOI:** 10.3389/fphar.2024.1472733

**Published:** 2024-12-10

**Authors:** Wensi Hu, Tongyao Li, Yu Du, Mingyang Yang, Si Liu, Songbai He, Qian Long, Xing Fan, Zinan Zhou, Xiaoyuan Li, Junzhao Liu

**Affiliations:** ^1^ Department of Critical Care and Emergency Medicine, West China School of Public Health, West China Fourth Hospital, Sichuan University, Chengdu, Sichuan, China; ^2^ Health Emergency Management Research Center, West China School of Public Health and West China Fourth Hospital, China-PUMC C.C. Chen Institute of Health, Sichuan University, Chengdu, Sichuan, China

**Keywords:** ethylicin, upper gastrointestinal bleeding, mucosal erosion, multiorgan injury, gastroscopy, esophageal stenosis

## Abstract

Ethylicin is a pesticide with excellent bactericidal ability. The incidence of poisoning has increased in recent years with the widespread use of ethylicin in green agriculture, but reports are lacking. In this study, we described three cases of oral ethylicin poisoning. Patients developed severe upper gastrointestinal bleeding after oral administration of ethylicin. Gastroscopy showed extensive mucosal erosions and ulcerations in the esophagus, stomach, and duodenum. Impaired consciousness, multiorgan injury, irreversible shock, and cardiac arrest were observed in cases where larger doses of ethylicin were ingested. Patients were treated with comprehensive therapeutic measures, including total gastrointestinal decontamination, medications such as proton pump inhibitors and somatostatin to reduce gastric bleeding. Endoscopic hemostasis was performed when pharmacologic hemostasis was not effective. Parenteral nutritional support and organ function support were given. In patients’ follow up, esophageal stenosis and dysphagia during feeding was noted, which severely affected the quality of life. Ethylicin poisoning has been a public health problem and the awareness should be raised.

## Introduction

**FIGURE 1:**
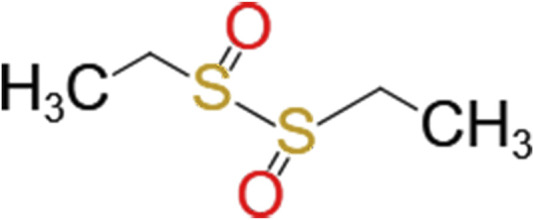
Formula of ethylicin.

Ethylicin is a broad-spectrum biomimetic fungicide and bactericide developed by the Shanghai Institute of Organic Chemistry of the Chinese Academy of Sciences. The chemical name of ethylicin is S-ethyl ethanethiosulfonate, and as shown in Figure 1, the molecular formula is C_4_H_10_O_2_S_2_ (CAS number 682-91-7) (Zhang et al., 2020). At room temperature, ethylicin appears as a colorless or slightly yellow emulsion and is soluble in various organic solvents. It has a strong garlic odor and is highly volatile (Huang et al., 2023).

Ethylicin is derived from allicin, which has a strong affinity with plants and can effectively combat various bacteria and fungi, suppress hyphal elongation, sporulation, and reduces the pathogenicity of *Phytophthora infestans* (Huang et al., 2023). It degrades rapidly and has few adverse effects for human and insect predators. In addition, ethylicin can also be used as a stimulator to promote plant growth. In recent years, ethylicin has been widely used in sectors such as grain, vegetables, cotton, and medicinal herbs with broad market prospects in China (Huang et al., 2023; Chen et al., 2012). However, ethylicin is highly irritating and corrosive to skin and mucous. Due to the widespread use of ethylicin, the incidence of oral ethylicin intoxication has been increasing, especially in South and Western South China, but relevant reports are lacking (Wang and Liang, 2020). In this study, we presented three cases of transoral ethylicin poisoning combined with severe gastrointestinal tract corrosion and bleeding, multiorgan injury and cardiac arrest, highlighting the serious toxicity to humans.

## Case presentation

### Case 1

A 52-year-old male with no known comorbidities presented to the emergency department complaining of nausea and vomiting for 10 h after ingesting ethylicin. Before admission, the patient took approximately 50 mL of ethylicin orally and then experienced recurrent nausea and vomiting of gastric contents with blood and food content in the vomitus. The family member transferred the patient to a local hospital, where the patient vomited about another 500 mL of bloody contents, his blood pressure was about 85–90/50–60 mmHg. He was given 80 mg of the proton pump inhibitor omeprazole intravenously, followed by a maintenance dose of 8 mg/h, rapid fluid resuscitation, and was transferred to our hospital for further management. Vital signs on admission were as follows: heart rate: 142 beats/min, respiratory rate: 31 breaths/min, blood pressure 97/71 mmHg. The patient was agitated, vomiting frequently, physical examination revealed that the extremities were cyanotic and cold. The laboratory tests showed that the white blood cell count and activated partial thromboplastin time were elevated (Table 1, Case 1). Computed tomography (CT) of the chest and abdomen showed extensive swelling and thickening of the gastrointestinal tract and patchy ground-glass opacities in the bilateral lungs. The patient was transferred to the intensive care unit for monitoring and management. He was treated with mechanical ventilation and rapid fluid resuscitation, continuous intravenous infusion of propofol emulsion (0.83 mg/kg/h) was administered for sedation, blood pressure was maintained with mesalamine (0.56 μg·kg^−1^·min^−1^) and norepinephrine (0.40 μg·kg^−1^·min^−1^). The patient was treated with continuous intravenous somatostatin (250 μg/h), omeprazole (8 mg/h), cefoperazone and sulbactam (3 g, every 8 h), and fresh frozen plasma of 600 mL was administered 4 h after admission. Owing to the patient’s inability to ingest food orally, parenteral nutrition was initiated on the day of admission, comprising a formulation of fat emulsion, amino acids, and glucose, with a total energy provision of 900 kilocalories.

**TABLE 1:** Laboratory test results of patients.

Laboratory tests	Case 1			Case 2		Case 3			Reference value
	Day 1	Day 3	Day 5	Day 1	Day 3	Day 1	Day 3	Day 11	
WBC (×10^9/L^)	22.39	16.99	14.73	35.45	12.82	12.02	8.02	5.6	4.0–11.0
RBC (×10^12/L^)	6.16	4.13	3.02	5.31	2.9	3.7	3.95	3.64	4.0–5.3
HGB (g/L)	197	137	97	170	93	114	121	110	114–154
PLT (×10^9/L^)	159	89	101	91	44	176	174	280	150–407
AST (U/L)	50	473	183	41	10,098	20	N	24	10–31
ALT (U/L)	19	554	387	35	6,897	12	N	57	6–29
LDH (U/L)	335	N	361	718	>53,750	224	N	141	120–250
TBIL (mmol/L)	25	10	19	19.1	41.3	18	N	12	0–21
ALB (g/L)	22.1	25.6	30.5	25.1	31.8	39.8	N	39.5	42–56
BUN (mmol/L)	10.49	13.48	N	5.66	2.36	4.86	N	N	2.6–6.5
Cr (μmol/L)	147	94	N	86	66.3	32	N	N	39–76
MYO (ng/mL)	356.5	N	N	80.6	>40,000	71.6	N	N	0–61.5
CK-MB (ng/mL)	117	N	N	2.72	217	35	N	N	0–24
TNI (ng/mL)	0.409	N	0.047	0.123	11.6	<0.012	N	N	0–0.034
PT (s)	15.7	N	12.6	13.5	45.4	12.2	N	N	9.6–12.8
PT-INR	1.49	N	1.18	1.23	4.13	1.07	N	N	0.88–1.15
APTT (s)	48.5	N	40.6	32.3	43.8	25.3	N	N	25.4–38.4
PH	7.205	7.358	7.425	7.13	7.51	7.37	7.43	N	7.35–7.45
K^+^ (mmol/L)	5.97	3.9	4.06	3.7	6.2	3.91	3.94	N	3.5–5.3
Na^+^ (mmol/L)	139.4	140.4	145.2	136	134	139.9	133.5	N	136–146
LAC (mmol/L)	5.73	1.65	0.9	6.9	11.9	1.63	0.93	N	0.36–0.75
PCT (ng/mL)	2.12	20.36	9.44	N	N	<0.1	N	0.25	0.0–0.5

WBC, white blood cells; RBC, red blood cells; HGB, hemoglobin; PLT, platelets; AST, aspartate aminotransferase; ALT, alanine aminotransferase; LDH, lactate dehydrogenase; TBIL, total bilirubin; ALB, albumin; BUN, blood urea nitrogen; Cr, creatinine; MYO, myoglobin; CK-MB, creatine kinase-MB; TNI, cardiac troponin I; PT, prothrombin time; PT-INR, prothrombin time-international normalized ratio; APTT, activated partial thromboplastin time; PH, potential of hydrogen; K^+^, serum potassium; Na^+^, serum sodium; LAC, lactate acid; PCT, procalcitonin; N, not examined.

**FIGURE 2:**
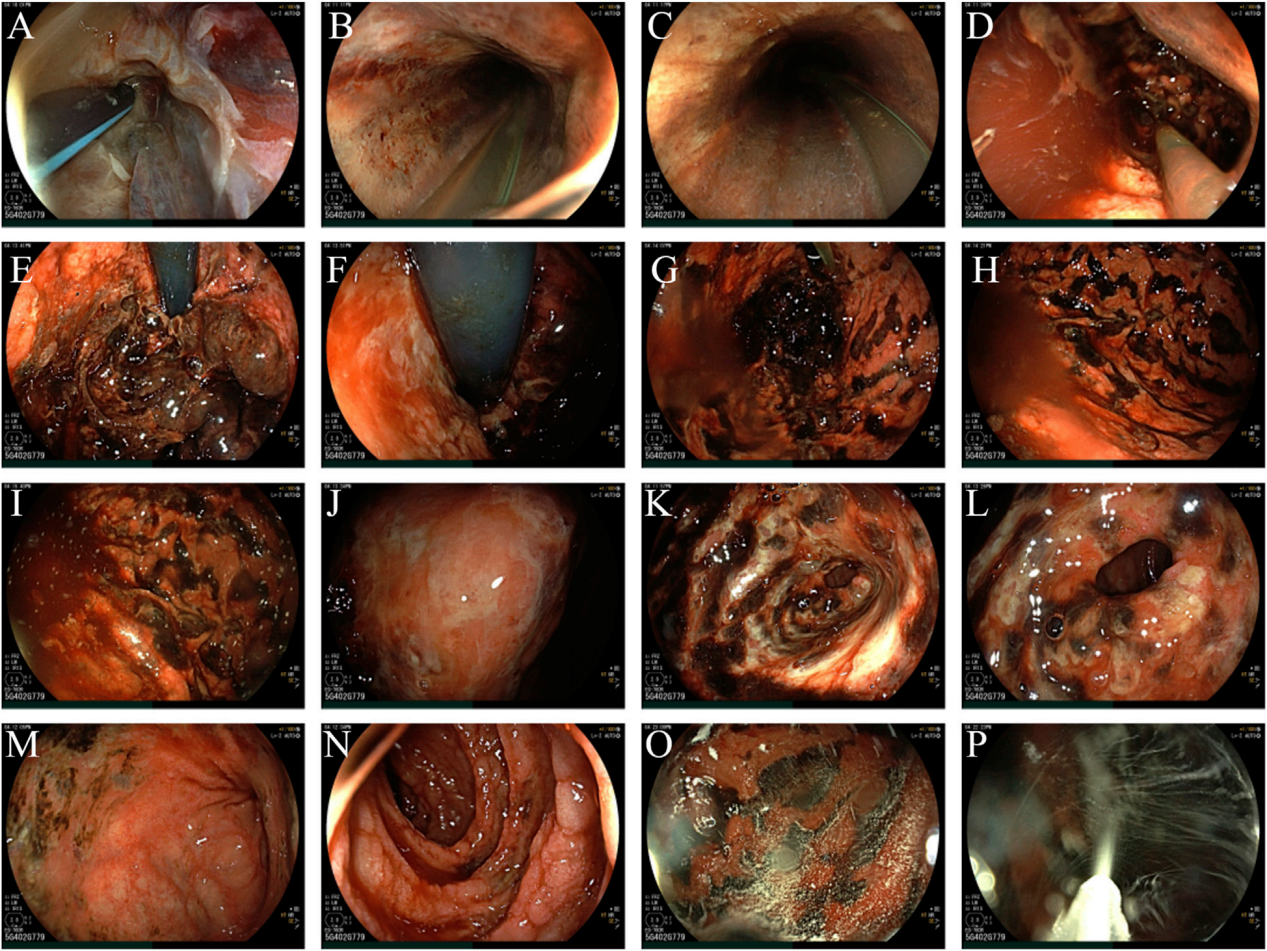
**(A)** lower pharynx; **(B)** upper segment of esophagus; **(C)** middle segment of esophagus; **(D)** lower segment of esophagus; **(E)** fundus ventriculi; **(F)** preventriculus; **(G)** corpus ventriculi; **(H)** gastric greater curvature (Zhang et al., 2020); **(I)** gastric greater curvature (Huang et al., 2023); **(J)** gastric angel; **(K)** sinuses ventriculi; **(L)**: pylori; **(M)**: duodenal bulb; **(N)**: descending part of duodenum; **(O)**: spraying of icy normal saline containing norepinephrine (Zhang et al., 2020); **(P)**: spraying of icy normal saline containing norepinephrine (Huang et al., 2023).

On the second day after admission, approximately 600 mL of bloody stomach contents and blood clots were leaked from the stomach tube. Therefore, bedside gastroscopy was performed. Extensive corrosions and ulcers with bleeding were observed in the lower pharynx, esophagus, stomach, and duodenum. Icy normal saline containing norepinephrine (concentration of 0.008%) was sprayed on the corrosions and ulcers to stop the bleeding (Figure 2). Two days after admission, a CT scan of the abdomen showed increased swelling of the gastrointestinal lumen, inflammatory changes and abdominal fluid in the abdominal cavity. On the third day of admission, there was a decrease in the amount of bloody stomach contents aspirated through the stomach tube. The tracheal tube was removed and mechanical ventilation was stopped on the fifth day of hospitalization. Vasopressors were discontinued to maintain blood pressure. The patient was discharged from the hospital on the seventh day for rehabilitation.

**FIGURE 3:**
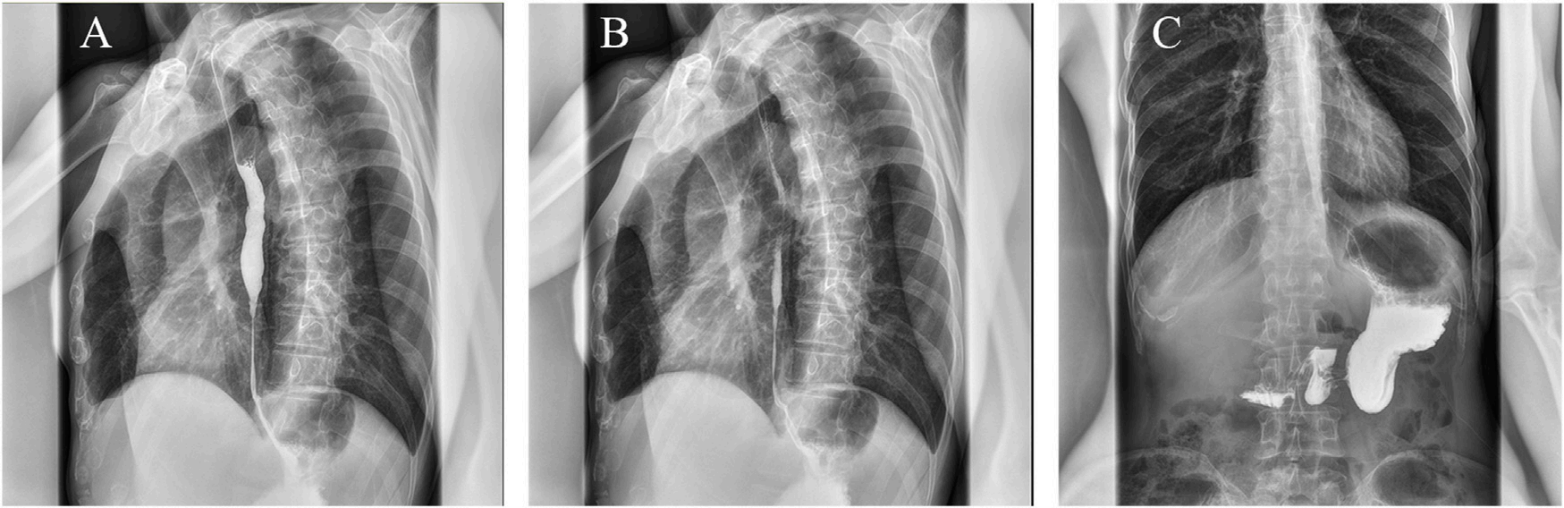
The sign of esophageal stenosis was seen on the barium X-ray. **(A–C)** The mucosa of the middle and lower esophagus is not smooth, and the lumen of the lower esophagus is narrowed.

Three months later, the patient experienced dysphagia, retrosternal pain, and vomiting while eating, so he visited the gastroenterology department of our hospital for treatment. Esophagography showed that the mucosa of the middle and lower esophagus was not smooth. The lumen of the lower esophagus (at the level of the nineth thoracic vertebra) was narrowed and the passage of contrast was slow (Figure 3). Gastroscopy was performed and showed esophageal stenosis with esophageal ulcer formation. Considering the high risk of esophageal bleeding and perforation, esophageal dilatation or stenting was not performed. The patient was instructed to follow a liquid diet and regular follow-ups.

### Case 2

A 66-year-old female patient presented to the emergency department (ED) with a reported history of ingesting 100 mL of ethylicin orally 6 h prior to admission. Approximately 10 min after oral ingestion, the woman was found unconscious and immediately transferred to a local hospital for gastric lavage. During gastric lavage, the patient vomited a large amount of bloody gastric contents. Immediately after the gastric lavage, the patient was transferred to our hospital for further treatment.

**FIGURE 4:**
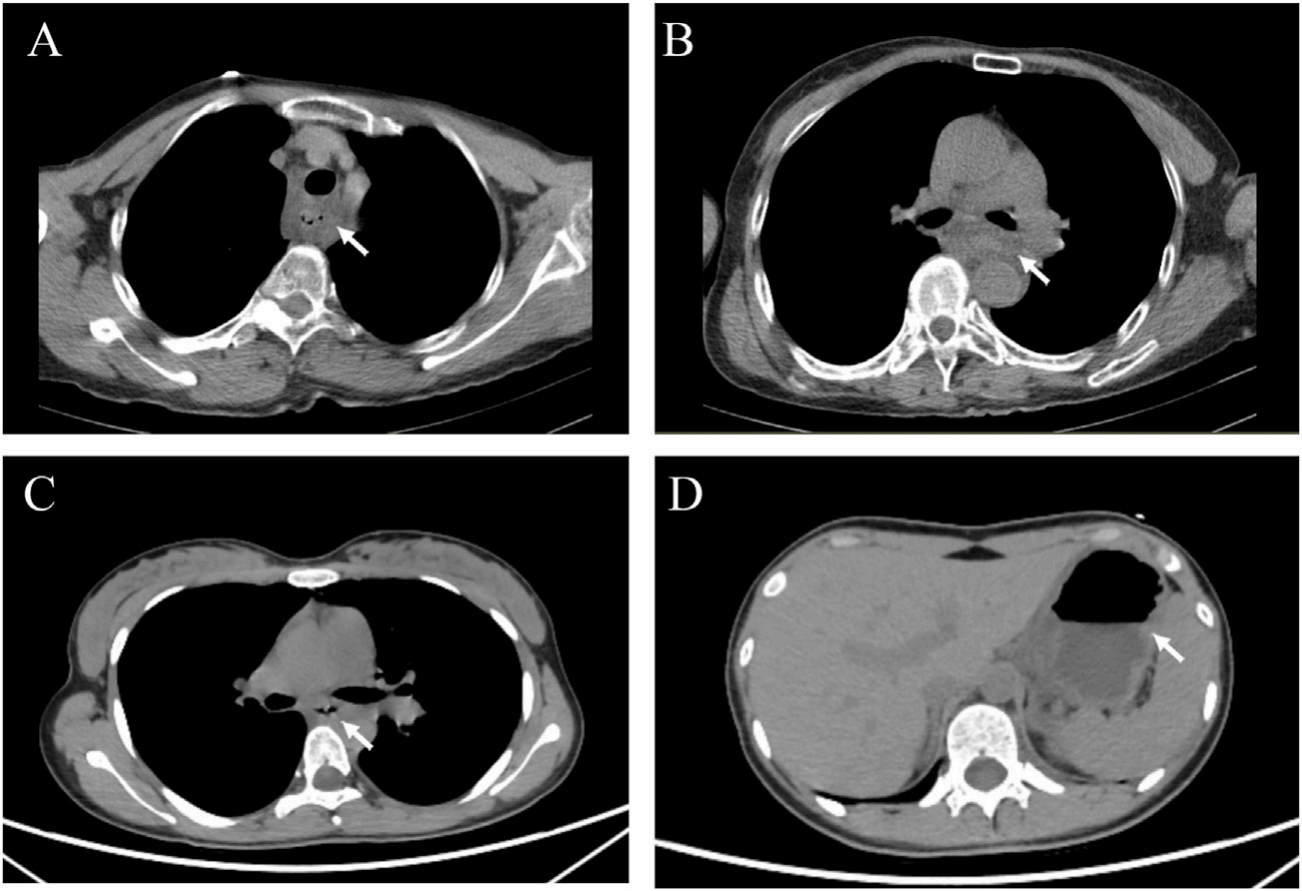
The computed tomography (CT) for patients in case 2 and case 3. **(A, B)** CT for the patient in case 2; **(C, D)** CT for the patient in case 3. The white arrows indicated the extensive swelling and thickening of the esophageal and gastric lumen.

At the time of admission, the patient was unconscious, a distinct garlic odor could be detected around the body, the extremities were cold with weak peripheral arterial pulsations. The results of laboratory tests showed the white blood cell count was elevated, while the concentration of albumin was below the normal range (Table 1, Case 2). CT scan showed extensive swelling and thickening of the esophageal and gastric lumen (Figures 4A, B). Mechanical ventilation was started, large volumes of fluids were infused, and continuous veno-venous hemofiltration (CVVH, Prismaflex, with low molecular weight heparin for anticoagulation) was performed to eliminate ethylicin. Omeprazole (8 mg/h), somatostatin (250 μg/h), glutathione (3.6 g daily), piperacillin sodium and tazobactam (3 g, every 8 h a day), and norepinephrine (0.60 μg·kg^−1^·min^−1^) were administered intravenously. On the second day after admission, the heart rate was over 140 beats/min, blood pressure was 99/68 mmHg, maintained with norepinephrine at a dose of 1.4 μg·kg^-1^·min^−1^, and CVVH was performed. Results of laboratory tests showed acute hepatic and cardiac injury, and coagulopathy. Bedside ultrasound examination revealed the left ventricular contractile function decreased significantly. On the third day, the patient’s condition continued to deteriorate, the lactate acid level increased to 11.9 mmol/L and cardiac arrest occurred, the relatives decided to terminate the treatment and pick up the patient from the hospital.

### Case 3

A 17-year-old female presented to our hospital 20 h after oral ingestion of ethylicin. Approximately 20 hours before presentation, the female ingested 5 mL of ethylicin, and then occurred vomiting accompanied by persistent burning pain in the pharynx and upper abdomen. The patient visited a local hospital and received emergency treatment. During the gastric lavage, the woman presented with nausea and vomiting of bloody gastric contents and was transferred to our hospital. On arrival, the patient was agitated and physical examination was unremarkable. The results of laboratory tests are shown in Table 1, Case 3. Chest and abdominal CT scans showed that the inner layer of esophagus and stomach was swollen (Figures 4C, D). The woman required suspension diet and treated with parenteral nutrition support, esomeprazole (intravenous administration of 80 mg and maintained at 8 mg/h), somatostatin (250 mg/h), and sucralfate suspensoid gel (1 g, twice daily). Due to significant swelling of the esophagus and stomach, gastroscopy was not performed to avoid increasing the risk of gastrointestinal bleeding or even perforation. The patient was discharged from the hospital after 10 days. The patient had a 3-month follow-up and was able to have a normal diet and had no impact on her life.

## Discussion

The main component of ethylicin is S-ethyl thiosulfinate, a derivative of allicin. Allicin has antimicrobial activities, including antifungal, antiviral, and antibacterial activity; however, the chemical structure is unstable, and the minimum inhibitory concentration is relatively high compared to amphotericin B and penicillin (Rauf et al., 2022; Qi et al., 2019). Ethylicin retains the structure of the active group of allicin and has better stability and antibacterial activity than allicin (Qi et al., 2019). Ethylicin has been increasingly used in green agriculture in recent years due to its basically harmless properties to other plants or insects. With the widespread use of ethylicin, there has been a gradual increase in the number of poisonings caused by ethylicin. In this study, we present three cases of patients with acute oral ethylicin poisoning. As with other toxins, the severity of ethylicin poisoning is dose-dependent. Ethylicin is highly corrosive and causes ulceration of the digestive tract mucosa and bleeding, with a risk of gastrointestinal perforation. In the three cases, typical symptoms and comorbidities include multiple corrosions and bleeding in the digestive tract. More seriously, ethylicin can cause consciousness alteration, multiple organ injury, irreversible shock, and cardiac arrest. In case 1, the patient appeared signs of altered consciousness and shock after oral administration of ethylicin, requiring endotracheal intubation and mechanical ventilation for respiratory support. In case 2, the patient ingested 100 mL of ethylicin orally in a large dose, after which he rapidly developed unconsciousness and shock. Despite aggressive treatment, laboratory tests showed rapidly elevated transaminase, creatinine, and lactic acid levels, indicating acute hepatic and renal injury. The multiorgan injury and metabolic acidosis was difficult to correct, eventually leading to cardiac arrest. The woman in case 3 ingested only 5 mL of ethylicin orally and showed no signs of altered consciousness, shock, or acute organ injury except for the presence of upper gastrointestinal bleeding. Similarly, two other reports described cases of transoral ethylicin poisoning that manifested as acute gastrointestinal corrosion, bleeding, coma, shock, and cardiac arrest (Wang and Liang, 2020; Shen, 2006).

There is no efficient study on the toxicological mechanisms of ethylicin in animal or human. One of the toxicology of ethylicin is the -S-S (=O)_2_ in the molecular structure, which can interfere with the metabolism of bacterial cells (Chen et al., 2012). Existing results from cellular and fungal experiments show that ethylicin induces the production of reactive oxygen species (ROS), which not only induces apoptosis, but also promotes the production of inflammatory cytokines such as interleukin (IL)-6, IL-8, and tumor necrosis factor-α (TNF-α), leading to immune-related damage in organs (Liu et al., 2023; Wang et al., 2023). Another study showed that ethylicin interfered with amino acid metabolism and stimulated the overexpression of apoptosis-inducing factors (Zhang et al., 2018). Huang et al. conducted a study on rats using 80% ethylicin emulsion and found that inhalation of ethylicin caused toxicity to the nervous and respiratory systems (Huang et al., 2013). At present, there is no case of poisoning caused by inhalation of ethylicin.

Many kinds of pesticides can cause esophageal and gastrointestinal mucosa corrosion after oral ingestion, such as paraquat and organophosphate pesticides. Paraquat induces cellular damage through oxidative stress and lipid peroxidation. It causes extensive multiorgan damage but predominantly accumulates in the lungs, leading to lung injury and respiratory failure (Tao et al., 2021). Organophosphorus pesticides can be absorbed through oral ingestion, inhalation, and skin and mucosal contact. They exert profound inhibitory effects on cholinesterase activity, which manifests as symptoms such as hyperhidrosis, sialorrhea, pulmonary rales, tremors, muscle weakness, with potential progression to respiratory insufficiency and death (Gupta et al., 2018). Unlike paraquat and organophosphorus pesticides, ingestion of ethylicin results in relatively minor damage to the lungs, liver, and kidneys. However, it has a severe corrosive effect on the esophagus and stomach. In severe cases, this can lead to gastricintestinal bleeding and esophageal stenosis. There is no specific antidote or guidelines for the treatment of ethylicin poisoning. Based on clinical experience, comprehensive treatment includes hemostasis, fluid replacement, protection of organ function, suspension of food, and parenteral nutritional support. The proton pump inhibitors omeprazole and esomeprazole, somatostatin, and mucosal protective agents such as sucralfate suspension gel can be given to alleviate the upper gastrointestinal bleeding (Costable and Greenwald, 2021). In the early stages following oral ingestion of ethylicin, gastric lavage may be performed in the absence of overt gastric bleeding. However, when definite gastric bleeding exists, gastric lavage is not recommended. In our report, patients experienced worsening gastrointestinal bleeding during gastric lavage at the local hospital. Milk and raw egg can be taken orally to protect the mucous of the digestive tract. Whole gastrointestinal cleaning plays an important role in comprehensive treatment. Medical charcoal tablets, montmorillonite powder and mannitol can be used for toxin absorption and induce diarrhea. Gastroscopy may increase the risk of worsening gastrointestinal bleeding, and the benefits and risks need to be fully assessed. When gastrointestinal bleeding is severe and pharmacologic hemostasis is not satisfactory, endoscopic hemostatic therapy can be used (Laine et al., 2021). As shown in case 1, gastrointestinal bleeding was effectively controlled after norepinephrine was sprayed through the gastroscope. Ethylicin ingestion has been associated with acute liver injury, necessitating the use of hepatoprotective agents like glutathione, which scavenges free radicals, mitigates cellular damage, and has demonstrated efficacy in attenuating hepatocyte toxicity induced by a spectrum of toxins, including ethylicin (Chen et al., 2013). Notably, patients may be at risk for aspiration pneumonia secondary to hematemesis, agitation, or gastric lavage. Consequently, potential pulmonary infections must not be overlooked, and antimicrobial therapy should be initiated as clinically indicated. Patients recovering from ethylicin poisoning with dysphagia should follow a diet of soft foods. If necessary, gastroscopy can be performed to assess the extent of esophageal stenosis.

## Conclusion

With the increasing use of ethylicin in agriculture, the incidence of ethylicin poisoning has increased and become a public health concern. Ethylicin causes severe gastrointestinal corrosion and bleeding, and excessive ingestion of ethylicin can lead to dysfunction of major organs. Public awareness of ethylicin intoxication should be increased.

## Data Availability

The original contributions presented in the study are included in the article/supplementary material, further inquiries can be directed to the corresponding author.
